# Supplementary material for: Chromothripsis during telomere crisis is independent of NHEJ, and consistent with a replicative origin
Source: Genome Res. 2019 May;29(5):737–49. doi: 10.1101/gr.240705.118 (PMC6499312; doi:10.1101/gr.240705.118)
Supplement: Supplemental Material [file supp_gr.240705.118_Supplemental_file_1.zip › contigs/annotated_contigs/DB47/contig.3.DB47_length_487_mean_cov_6.27104722793.docx]

**DB47_length_487_mean_cov_6.27104722793**

```
CGGGTGAATGACGGCAGCTGTGCCAATGGGAATAGGCTACCTGGGAGCCATGCGTGTACAACATGCAGGCTTGCTCCATGTTCCCTCTT
 >chr19:6885358-6885575 + E=6e-101 p=5e-03
CTTTATCAACCACGTGTACGGTAAAGGAACAGGCAACACGGTCCTGGCCAGGTAGAGACCCCATCTGCATAATAAAATATTAGGGTGGG

CAGCCAGCTTCTTTGTACTCTATGTAAACGGCACACCTG|C|CTTTCTAGCTCCCGTATCAGCGTTAAGCTAGGGCATATTAATGTGTT
                                         >chr19:6884728-6884897 - E=4e-90
GTTAGGTTATGGGTGTGGGCAGCCACTGATAGCAGCTGTCACAAGGTACAAAGGAGGTGCATAGAACATCCCCGAATTCTTAGAAAGAA

AAGCTCCACGTGTTTTTGCCTTCCCCTTTTGT|T|TTCTTTCTTTCTTTTTTTTTTTTTTGAGATGGAGTCTTGCTCTGTCACCCAGGC
                                 >chr19:6885740-6885840 + E=2e-44
TGGAGTTCAATGGCACAATCTCGGCTCACTGCAACCTCTGCTTCCC
```
